# Experimental Investigation on the Static Performance of Stud Connectors in Steel-HSFRC Composite Beams

**DOI:** 10.3390/ma14112744

**Published:** 2021-05-22

**Authors:** Fangwen Wu, Wenlong Tang, Chengfeng Xue, Guorui Sun, Yanpeng Feng, Hao Zhang

**Affiliations:** 1 School of Highway, Chang’an University, Xi’an 710064, China; wufangwen@chd.edu.cn (F.W.); 2019221004@chd.edu.cn (W.T.); 2019221027@chd.edu.cn (Y.F.); 2 School of Civil Engineering, Xijing University, Xi’an 710123, China; xuechengfeng@xijing.edu.cn; 3 School of Civil Engineering, Harbin Institute of Technology, Harbin 150090, China; 19B933012@stu.hit.edu.cn; 4 State Key Laboratory of Mechanical Behaviour and System Safety of Traffic Engineering Structures, Shijiazhuang Tiedao University, Shijiazhuang 050043, China

**Keywords:** high strength fiber reinforced concrete (HSFRC), stud connector, static performance, push-out test, stud dimension

## Abstract

In this research, high strength fiber reinforced concrete (HSFRC) was used to replace the normal strength concrete (NSC) in steel-concrete composite beams to improve their working performance, which might change the static performance of stud connectors. Firstly, push-out tests were conducted to investigation on the static performance of stud connectors in steel-HSFRC composite beams and compared with steel-NSC composite beams. Studs of 8 sizes, 13 mm, 16 mm, 19 mm and 22 mm in diameter and 80 mm and 120 mm in height were adopted to study the influence of stud dimension. The test phenomenon shown that the crack resistance of HSFRC was better than that of NSC, and there were some splitting cracks on NSC slabs whereas no visible cracks on HSFRC slabs when specimens failed. Next, the load-slip curves of studs were analyzed and a typical load-slip curve was proposed which was divided into four stages. In addition, the effects of test parameters were analyzed according to the characteristic points of load-slip curve. Compared with NSC slab, HSFRC slab could provide greater restraining force to the studs, which improved the shear capacity and stiffness of studs while suppressed the ductility of studs. The shear capacity, stiffness and ductility of studs would significantly increase with the increasement of stud diameter and the studs with large diameter were more suitable for steel-HSFRC composite beams. The stud height had no obvious influence on the static performance of studs. Finally, based on the test results, the empirical formulas for load-slip curve and shear capacity of stud connectors embedded in HSFRC were developed which considered the influence factors more comprehensively and had better accuracy and applicability than previous formulas.

## 1. Introduction

In recent years, the steel-concrete composite beams have been widely used in engineering structure because of the superior performance and the better comprehensive benefits [1,2]. In steel-concrete composite beam, the shear connector bears the function of transmitting the longitudinal shear force between the steel beam and the concrete slab [3]. Due to the complex stress state, the shear connectors are vulnerable to damage [4]. The headed stud is the most commonly used shear connector thanks to its convenient construction and low price [5]. Therefore, in order to ensure the safety of steel-concrete composite beams, researchers all over the world have paid attention to the static performance of stud connectors [6,7,8].

Generally, concrete slabs of steel-concrete composite beams are made of NSC, so there is muc research on the static performance of studs in steel-NSC composite beams. Through push-out test, Buttry et al. [9] indicated that the shear capacity of studs embedded in NSC was primarily influenced by the compressive strength and the elastic modulus of concrete and creatively proposed the empirical expression of shear capacity and the load-slip curve of studs. After that many scholars also conducted to more in-depth studies on the behaviour of studs in steel-NSC composite beams and shown that that the shear capacity of stud connectors increased with the diameter of studs while the height of stud had no obvious influence on that [10,11]. In addition, some empirical formula for load-slip curves and shear capacity of studs considering stud dimension and concrete properties were developed [12,13]. At present, the research on the static performance of studs in steel-NSC composite beams has been mature, and some mainstream codes have given the calculation methods of shear capacity of stud embedded in NSC [14,15,16].

However, it has been found that in the practical use of steel-NSC composite beams, such as cracking of concrete slab in negative moment area, poor fatigue resistance, weak durability in extreme environment and so on [17]. A number of researchers proposed to replace NSC with fiber reinforced concrete (FRC) and ultra-high-performance concrete (UHPC) in order to enhance the working performance of steel-concrete composite beams [18]. After the replacement of the material of concrete slabs, the static performance of the stud connectors would also change accordingly. Zhang et al. [19] through tests found that compared with steel-NSC specimens, the shear capacity of the studs embedded in FRC was slightly improved, the crack resistance of FRC slab was better and the failure mode would change when the stud diameter exceeded 23 mm. He et al. [20] concluded that the shear capacity and ductility of the stud connectors embedded in FRC would increase with the fiber volume fraction studied by push-out tests and finite element analysis. For the steel-UHPC composite beams, their failure mode was different from that of steel-NSC composite beams during push-out test [21]. The shear capacity of stud connectors embedded in UHPC slabs would increase significantly, but the ductility would decrease too much to meet the requirements of relevant specifications [22]. In addition, the stud diameter had significant on the static behavior of studs in UHPC, and short and large studs were more suitable for steel-UHPC composite beams [23].

Even though replacing NSC with FRC and UHPC would improve the working performance of steel-concrete composite beams to a certain extent, it still has some limitations. The compressive strength of FRC was similar to that of NSC, so the performance improvement of steel-FRC composite beam was not very great [20]. The UHPC slab is great more expensive than NSC slab, and faces the problems of complex construction technology, strict maintenance condition, large shrinkage and so on. Furthermore, the strength of UHPC could not be fully utilized in steel-concrete composite beams [23,24]. In view of these problems, using HSFRC instead of NSC in steel-concrete composite beam might be a better option. Since HSFRC is a new type of concrete whose performance improved by adding superplasticizer, mineral admixtures and steel fiber. Its compressive strength is usually 80~150 MPa and axial tensile strength is 10~20 MPa [25]. In addition, the energy absorption capacity and durability of HSFRC were better than NSC and FRC. The price of HSFRC was lower than UHPC, and the construction and maintenance were more convenient [26]. Moreover, different fracture toughness of various concrete will result in different failure modes [27]. At present, HSFRC was mainly used in special protection structures and structure-strengthening engineering [28,29]. However, there was hardly research on steel-HSFRC composite beams. Hence, it is essential to study the static performance and of stud connectors embedded in HSFRC and the influence factors on it (e.g., stud diameter and height and concrete property) for increasing the understanding of steel-HSFRC composite beams.

In order to research the static performance of stud connectors in steel-HSFRC composite beams, 4 steel-NSC and 8 steel-HSFRC push-out specimens with different dimensions of studs were tested. Firstly, the failure modes of specimens and the shear capacity, stiffness and ductility of stud connectors were described and analyzed. Next, in accordance with the load-slip curves, the influence law of concrete type, diameter and height of studs on the static performance of stud connectors was revealed. Finally, empirical formulas for load-slip curve and shear capacity of stud connectors in steel-HSFRC composite beams were developed by test results fitting. This study provided a new method to improve the performance of steel-concrete composite beams. In addition, the mechanical behavior of steel-HSFRC composite beams was studied in detail, and the relevant calculation formulas were proposed. The research results could provide reference for the design and application of the new type of composite beam.

## 2. Experimental Program

### 2.1. Design of Specimens

**Figure 1:**
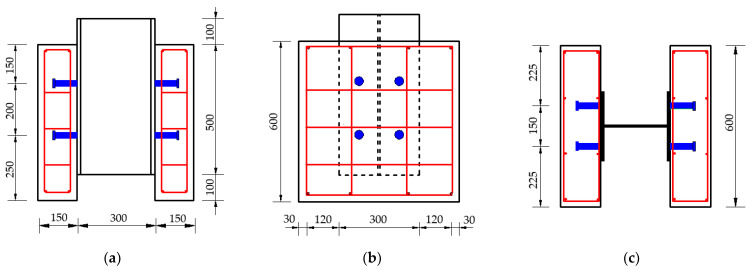
Details of specimens: (**a**) front view; (**b**) side view; (**c**) top view (unit: mm).

A total of 12 composite beam specimens were prepared in this push-out test to investigate the effect of concrete type and the diameter and height of headed stud on the static behavior of stud connectors. According to Eurocode-4 [14] and model similarity principle, these specimens were designed and fabricated, as shown in Figure 1. Each specimen consisted of one H-shape steel girder, two concrete slabs and eight studs, and the configuration of all specimens was the same, except for the sizes of studs. H-shape steel with dimension of HW 300 × 300 × 10 × 15 [30] and studs of 8 sizes, 80 mm and 120 mm in height and 13 mm, 16 mm, 19 mm and 22 mm in diameter, were adopted in this research. The studs were fastened with H-shape steel girder by welding all around. To distribute the load evenly during loading, a 300 mm × 300 mm steel plate was welded on the top of the H-beam of the specimen. For the purpose of good welding strength, J506 electrode was used for welding. The surfaces of H steel were oiled to prevent the bonding force affecting the test results. These concrete slabs were reinforced by hot rolled ribbed bars with diameter of 8 mm. After the steel bars skeletons was bound, the templates were made. Next, the concrete slabs were casted by two types of concrete, NSC and HSFRC, respectively. Vibration was carried out at the same time as concrete was casting. After the completion of the specimens’ production, the specimens were put into a curing room with a temperature of 20 ± 2 °C and a relative humidity of ≥95%. After curing for 1 day, remove the templates, and then continue curing to 28 days for subsequent push-out test. Every specimen was assigned a unique specimen ID, and they and specific parameters of specimens are as shown in Table 1.

### 2.2. Material Properties

The NSC and HSFRC used in this test were commercial concrete directly purchased from Zhongde Xinya Building Materials Co., Ltd, Xinmi, China. The mixture proportions of NSC and HSFRC are summarized in Table 2. The main difference between NSC and HSFRC was that there was silica fume and steel fiber in HSFRC, but not in NSC. When the concrete slabs were being casted, each kind of concrete was sampled. According to CECS 13:2009 [31], three groups of 100 × 100 × 100 mm^3^ cubic specimens and 100 × 100 × 500 mm^3^ dog bone specimens were fabricated to test the compressive and tensile strengths of concretes. The elastic modulus of concretes was obtained by DT-2 Dynamic elastic modulus tester. The measured material properties of concretes were shown in Table 3.

The H-shaped steel girder and the steel plate at the top of the push-out specimen were made by Q345D steel, whose yield strength, tensile strength and elastic modulus were 345 MPa, 470 MPa and 209 GPa, respectively. The reinforcement bars in concrete slabs were HRB400 bar with diameter of 8 mm, and they yield strength, tensile strength and elastic modulus were 400 MPa, 470 MPa and 200 GPa, respectively. All stud connectors were grade ML15, but they had different dimensions. The mechanical properties of studs were provided by merchant, as shown in Table 4.

### 2.3. Test Setup and Instruments

**Figure 2:**
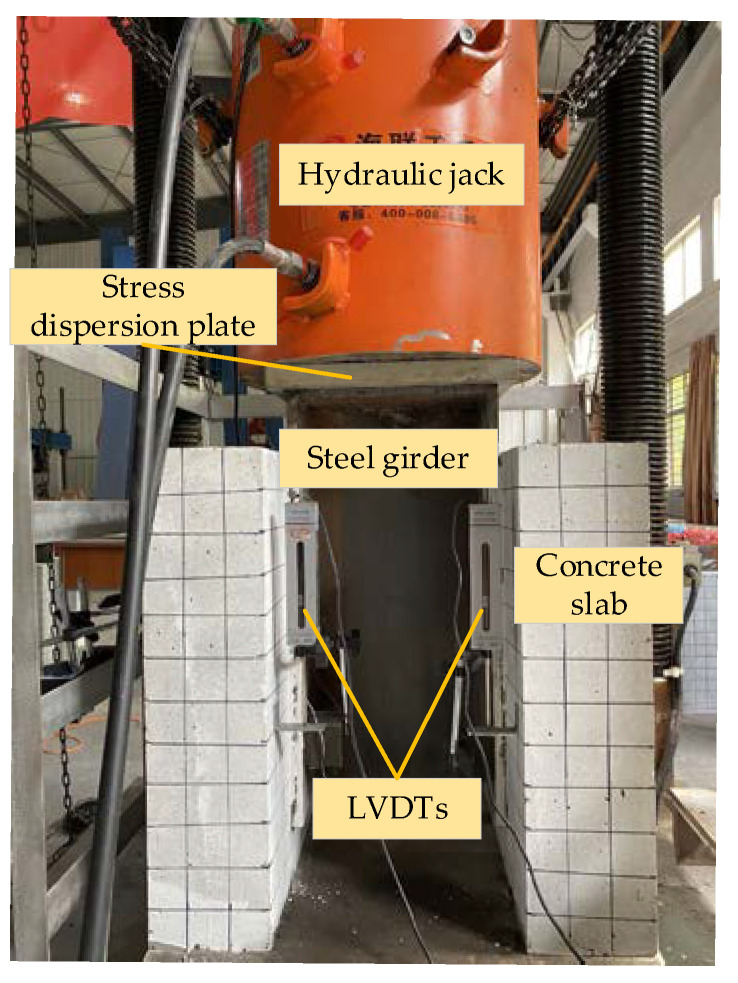
Test setup and specimen.

Figure 2 shows the test setup and specimen. It can be seen that 2 LVDTs were mounted on the concrete slabs, and there were also two LVDTs at the same position on the back. These LVDTs were fixed on the concrete slabs and connected to the steel girder to measure the interfacial relative slip between the steel girder and the concrete slabs. The specimen was placed on a steel plate and the bottom of the specimen was spread with fine sand. To transfer the load uniformly, the top steel plate was sanded flat, and a stress dispersion plate was placed between the load device and the specimen.

The push-out test was conducted by a microcomputer-controlled compression-shear fatigue test loading system (PLU-1000) with a load capacity of 1000 t. Before formal loading, the specimen was preloaded for 3 times with a load of 20% of the elastic bearing capacity obtained by theory and finite element calculation. During the preloading period, the influence of the nonlinear force and the gap between the various parts of the test system was reduced and the working conditions of loading system and the LVDTs were checked. After the end of the preloading 10 min, the data of each measuring point were recorded as the initial state of the test, and the formal test loading began. The specimen was loaded generally according to the increment of 15 kN, while the load increment was reduced to 5 kN when the specimen stepped into the elastoplastic critical stage. The loading rate was contained at 5 kN/min. After completion of every stage loading, the load was maintained for 5 min to make the specimen fully deformed. When the breakout of concrete and fracture of studs occurred, it was considered that the specimen had been damaged, and the test was finished. After the specimen failure, the failure surfaces of the specimen were observed, and the visible cracks on the concrete slab were marked. In the end, the concrete slabs were broken, and the studs were taken out to observe they deformation.

## 3. Test Results and Discussion

### 3.1. Failure Mode

**Figure 3:**
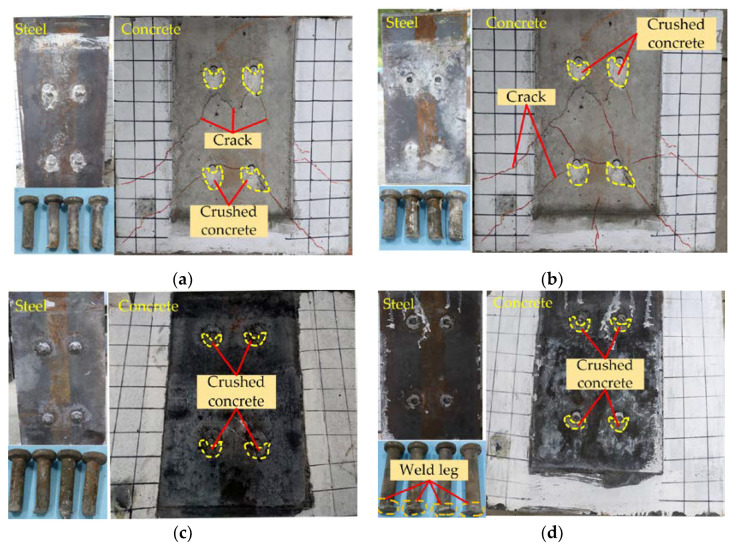
Failure modes of test specimens: (**a**) N80-16; (**b**) N80-22; (**c**) H80-16; (**d**) H120-22.

Some previous studies on push-out shown that there were three modes of failure of composite beams with stud connectors: concrete breakout, stud failure and combine failure of concrete and studs, among which stud failure can be divided into failure from shank and from weld [32]. In this test, the combined failure and stud failure the two failure modes were observed. The surfaces of H-shape steel, concrete slabs and studs of some representative specimens with the two failure modes are shown in Figure 3.

For the NSC specimens, the failure modes were combined failure of concrete and studs as shown in Figure 3a,b. During the loading process, some subtle cracks occurred on the NSC slabs, and these cracks continued to extend with the increase of load. The test ended with the studs being cut off, and at this time, there were some cracks on NSC slabs, but they are not completely breakout. The cracks on NSC slab were mainly diagonal cracks, with a small number of horizontal cracks and vertical splitting cracks. The maximum crack widths on N80-16 and N80-22 specimens were 3.2 mm and 4.8 mm, respectively. The largest cracks were both found under the studs on the left of the first row. It also can be seen that the number of cracks on H80-22 is more than that on H80-16. These phenomena indicated that the NSC slabs would be damaged before the stud fracture, and the larger the stud diameter was, the more serious the damage of the NSC slab would be.

It can be seen from Figure 3c,d that there is no visual crack occurred on the HSFRC slabs, and the same phenomenon also is observed in the other HSFRC specimens. Hence, the failure modes of HSFRC specimens were stud failure. It is because that HSFRC had high strength and cracking resistance, and the common studs were not enough to destroy it. In addition, due to the existence of steel fiber, the HSFRC slabs had better fracture toughness than NSC, and so HSFRC slabs could withstand large deformation without cracking [27]. This illustrated that the stud connectors with larger diameter and higher strength were more suitable for HSFRC-steel composite beam to give full play to the advantages of HSFRC. In addition, it is noted that the studs in H120-22 specimen fractured from weld, and those in the others HSFRC specimens all failed from the shank of stud. The reason for this phenomenon is that the HSFRC and the studs with large diameter and height both have high strengths which resulted the weld became weak point. Therefore, in the construction of composite beam, it is necessary to ensure that the weld have enough strength to prevent structural damage caused by the weld in use.

In addition, some common damage characteristics were observed in NSC and HSFRC specimens. There was a small area below every stud on all the concrete slabs where the concrete was crushed, but the crushed areas on HSFRC slabs were smaller than NSC slabs. This phenomenon indicated that the concrete below the stud would receive great compressive stress and the HSFRC had better damage resistance than NSC. It also can be found that all the studs only had a small deformation at the shank root, and the remaining parts of shanks embedded in the concrete slabs remain upright. It manifested that NSC and HSFRC both had a good embedding effecting on the studs, which limited the deformation of studs at a certain extent.

### 3.2. Load-Slip Relationship

**Figure 4:**
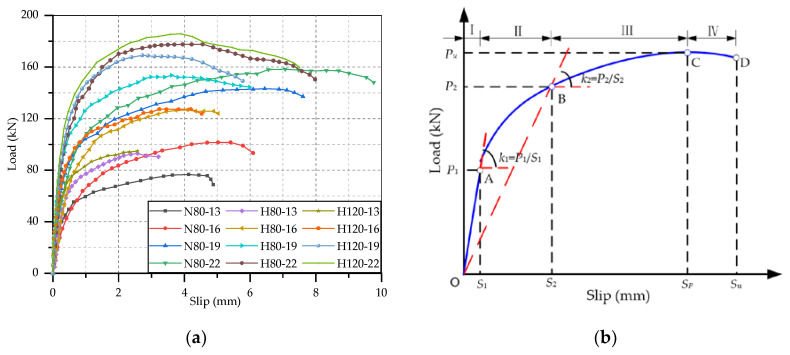
Load-slip curves of specimens: (**a**) test load-slip curves; (**b**) typical load-slip curve.

The load-slip curve is an important basis for analyzing the mechanical behavior of stud connector, and which could completely reflect the variation of mechanical performance of stud during the process of push-out test. The load-slip curves of all the push-out specimens in this test are shown in Figure 4a. The slip value is the average of four the readings of LVDTs, which represents the interfacial relative slip between H-shape steel and concrete slab. As can be seen that each curve has a similar trend, but there is a big difference in the positions of the vertex and the end point. This manifested that the steel-concrete composite beams with studs had the same deformation stages, which were hardly influenced by the type of concrete slabs and the dimension of studs. These curves rose very fast linearly at a small slip, and then the rising rate gradually decreased and tended to be flat. After these curves reached the peak point, the load began to decline slowly while the slip continued to increase until specimens failed. It could be concluded that these steel-concrete composite beams would appear large deformation before failure, and so the failure modes were ductile failure.

The load-slip curves were divided into four stages in according to the characteristic points and the deformation process of steel and concrete under stress [13,33]. In order to facilitate the expression of load-slip curve, an idealized load-slip curve of stud was drawn, as shown in Figure 4b. The idealized load-slip curve contained four stages, namely elastic deformation stage, yield stage, plastic strengthening stage and failure stage. These four stages were described and explained as follows:

- Elastic deformation stage (O-A): The load was proportional to the relative slip and the relative slip was quite small. Generally, the slope of the curve at this stage was taken as the initial shear stiffness of the stud. There was no visible change and crack in all the specimens. Since the studs and concrete slabs of specimens were in elastic stage, and the specimens did not reach normal use state.
- Yield stage (A-B): The slop of load-slip curve started to decrease, and it changed from a slanting straight line to a convex curve. The part of concrete in contact with the shank root entered the plastic deformation stage, which caused the lateral support of concrete to the stud to decrease gradually. With the continuous increase of load, the studs gradually yielded, and some cracks occurred in NSC slabs. It was these reasons that resulted in the increasement relative slip accelerating. The secant slope at the end point of this stage could be considered to be the average stiffness of the entire deformation process of the stud.
- Plastic strengthening stage (B-C): The rising rate of load-slip curve further decreased, and the curve gradually rose to the peak point and then tended to be stable. The concrete in a certain area under the stud root had completely crushed. The studs entered the plastic strengthening stage from the yield stage, during which their deformation increased continuously. In this stage, some diagonal and splitting cracks would occurr and developed on NSC slab, while there was no visible crack on HSFRC slabs. It could be seen that there was a significant displacement between the H-shape steel and the concrete slabs.
- Failure stage (C-D): The load on the stud decreased gently with increase in the relative slip after the stud reached the maximum. In the end, the load decreased sharply and suddenly, indicating the failure of the specimen. The load-slip curves here did not show the final steep decline section, because the specimens had failed at this time, which was not of research significance. All the specimens made a loud noise when they were broken, and the studs were cut off.

### 3.3. Effects of Test Parameter on Static Performance

**Figure 5:**
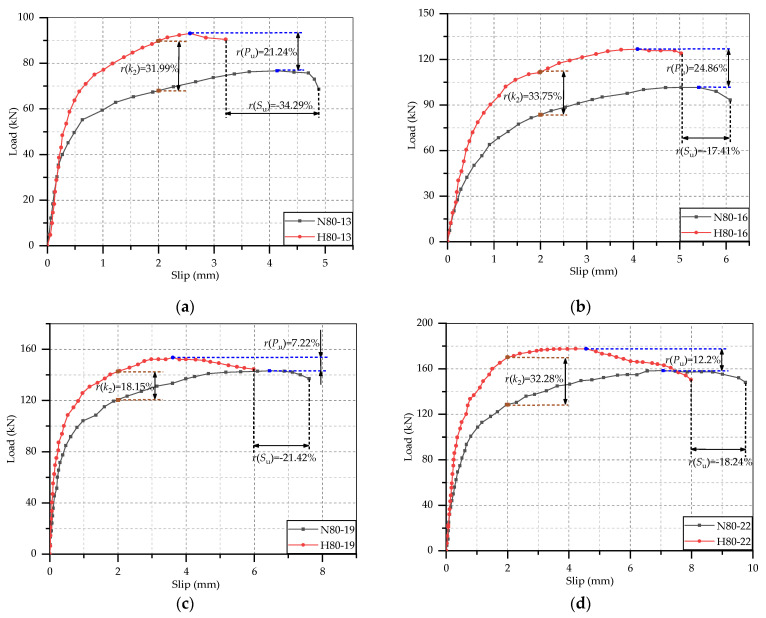
Effect of concrete type: (**a**) *d* = 13 mm, *h* = 80 mm; (**b**) *d* = 16 mm, *h* = 80 mm; (**c**) *d* = 19 mm, *h* = 80 mm; (**d**) *d* = 22 mm, *h* = 80 mm.

**Figure 6:**
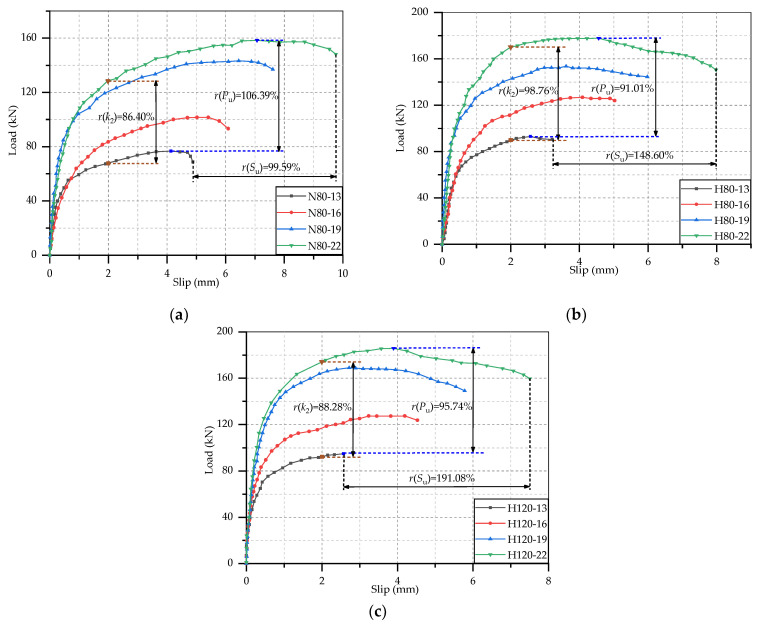
Effect of stud diameter: (**a**) N80-13~22; (**b**) H80-13~22; (**c**) H120-13~22.

**Figure 7:**
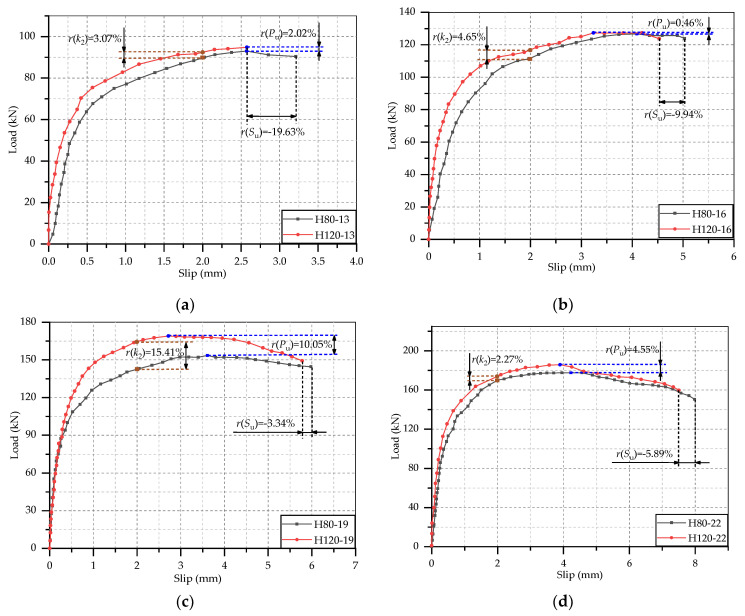
Effect of stud height: (**a**) *d* = 13 mm; (**b**) *d* = 16 mm; (**c**) *d* = 19 mm; (**d**) *d* = 22 mm.

These load-slip curves were processed, and test results were obtained to analyze the static performance of these studs. It was considered that each stud was subjected same force in the test, so the shear capacity of per stud was 1/8 the ultimate load of the specimen. According to the previous research, the push-out specimen was in the elastic deformation stage when the slip is 0.2 mm, and it was in the elastic-plastic deformation stage when the slip reaches 2 mm. Hence, the secant slope of the load-slip curve at the interfacial slip of 0.2 mm and 2 mm could be used to calculate the shear stiffness [32]. The ultimate load of push-out specimen, the shear capacity of per stud, the ultimate interfacial slip and the shear stiffness at slip of 0.2 mm and 2 mm were listed in Table 5. From this table, there was no significant regularity in the shear stiffness at slip of 0.2 mm, but some typical phenomenon could be found in the shear stiffness at 2 mm slip, which was similar to results of Qi et al. [32] and Wang et al. [23]. Hence, the variation rule of shear stiffness was analyzed by the shear stiffness out of 2 mm. In addition, in order to learn clearly the effects of test parameters on the static performance of studs, the comparisons of the load-slip curves of specimens with different test parameters are separately shown in Figure 5, Figure 6 and Figure 7, where *r*(*P*_u_), *r*(*S*_u_) and *r*(*k*_2_) represent the change rate of shear capacity, ultimate interfacial slip and shear stiffness of stud. Combined the load-slip curves with specific test results in Table 5, the effects of concrete type, stud diameter and stud height on the static performance of stud shear connector are given below.

#### 3.3.1. Effect of Concrete Type

It can be found from Figure 5 that the growth rate and maximum values of the load-slip curves of HSFRC specimens are both higher than those of NSC, and the HSFRC curves always end before the NSC curves. This suggests that in comparison with the stud in NSC slab, the shear capacity and stiffness of those of stud in HSFRC slabs were increased while the ductility was declined. The shear capacity of the studs with diameter of 13, 16, 19 and 22 mm in steel-HSFRC specimens, respectively, increased by 21.24%, 24.86% 7.22% and 12.2%, the shear stiffness, respectively, increased by 31.99%, 33.75%, 18.15% and 32.28%, but the ultimate slips, respectively, decreased by 34.29%, 17.41%, 21.42% and 18.24%. This is because HSFRC has better mechanical properties which could provide more binding force to the studs, but also effectively limit the deformation of the studs. In addition, all the NSC slabs cracked, and the bigger the stud was, the more serious the cracking was. However, there was no visible crack on the surface of HSFRC slab. Therefore, the use of HSFRC slab could better play the performance of the stud shear connectors and improve the shear capacity of the whole structure. It is worth noting that in the design of HSFRC-steel composite beam, focus should be paid on its ductility to ensure that can meet the requirements of the relevant codes.

#### 3.3.2. Effect of Stud Diameter

As can be seen from Figure 6 that the growth rate, height and traverse extension height of the load-slip curve increase significantly with the stud diameter. Hence, the increasement of stud diameter could notably improve the static performance of stud in term of shear capacity, ductility and shear stiffness. Take the specimens H80-13~22 as an example, the shear capacities of H80-16, H80-19 and H80-22 were, respectively, 36.29%, 65.12% and 91.01% higher than that of H80-13. In comparison with H80-13, The ductility of H80-16, H80-19 and H80-22, respectively, increased by 56.70%, 86.29% and 148.60%, and their shear stiffness, respectively, increased by 24.59%, 58.93% and 89.76%. The increase in shear capacity and stiffness of studs is due to the larger cross-sectional area of the studs with larger diameter. At the same time, large diameter stud would cause more serious damage to the concrete slabs, thus increasing the ultimate slip and the ductility. Therefore, in the construction of steel-concrete composite beams, large diameter stud shear connectors could be appropriately selected to improve the overall mechanical properties of the structure.

#### 3.3.3. Effect of Stud Height

The comparison of the load-slip curves of studs with different height embedded in HSFRC slabs is shown in Figure 7. It can be seen that the curves of 120 mm height studs are generally above the curves of 80 mm height studs, and the extension heights are slightly less than the curves of 80 mm studs. This phenomenon indicated that the increasement of stud height would slightly improve the shear capacity and stiffness of studs but would decrease the ductility of studs a little. Compared with the studs with a height of 80 mm, the shear capacity and stiffness of 120 mm height studs increased, respectively, by 4.27% and 6.35% on average, while the ultimate interfacial slip declined by 9.70% on average. It can be seen that the change rates of the three static performance indexes were all less than 10%. Therefore, when the aspect ratio of the stud was appropriate, it could be generally considered that the length of the stud has no effect on the static performance of the stud [21].

## 4. Evaluation of Test Results

### 4.1. Load-Slip Curve

The load-slip curve is the most important representation of the static shear behavior of stud connectors, which plays a significant role in analyzing the shear capacity, stiffness and ductility of studs. If the load-slip curve of stud could be correctly predicted by mathematical expression, the development of steel-concrete composite beam design and evaluation of the structural performance of existing similar structure would be greatly promoted. At present, based on the results of push-out tests, many researchers have put forward some equation to express the load-slip curve of stud connectors by mathematical fitting. Majority of these studies have focused on the studs embedded in NSC, and minority of them have given the equations for the stud in HPC and UHPC. However, there is little research has been studied on the studs in steel-HSFRC composite beam.

Ollgaard et al. [34] carried out continuous push-out test on 48 steel-NSC composite beam specimens and fitted a classical formula for predicting load-slip curve of stud connectors based on the test results. The load-slip curve formula is as follows:

(1) $$\frac{P}{P_{u}}={\left(1-e^{-18\cdot S}\right)}^{0.4}$$

where *P* represents the load on per stud; *S* represents the interfacial slip.

To study the influence of the different properties of concrete type, An and Cederwall [35] tested the shear performance of 4 steel-NSC and 4 steel-HPC push-out specimens. In addition, a non-linear regression was carried out on the experimental results, and the empirical expressions of the load-slip relationship of studs in steel-NSC and steel-HPC were, respectively, given by:

(2) $$\frac{P}{P_{u}}=\frac{2.24\cdot (S-0.058)}{1+1.98\cdot (S-0.058)}\;\mathrm{for}\;\mathrm{NSC}\;\mathrm{specimens}$$

(3) $$\frac{P}{P_{u}}=\frac{4.44\cdot (S-0.031)}{1+4.24\cdot (S-0.031)}\;\mathrm{for}\;\mathrm{HPC}\;\mathrm{specimens}$$

Xue et al. [12] conducted 30 push-out tests to study the effects of different parameters (e.g., stud diameter and height, concrete strength, welding technique and so on) on the static behaviors of stud connectors. Based on the test results, a more accurate expression of stud load-slip curve was put forward:

(4) $$\frac{P}{P_{u}}=\frac{S}{0.5+0.97\cdot S}$$

It can be seen that the stud diameter was not taken into account in the above expressions. However, it was found in this study that the stud diameter had a great influence on the variation trend of load-slip curve. Similarly, Wang et al. [23] also discovered the significant effect of stud diameter on the static behavior of stud connectors by testing tested 6 steel-NSC and 12 steel-UHPC push-out specimens. Hence, they proposed an empirical load-slip expression considering the stud diameter, and the expression could be applicable to both NSC and UHPC specimens. The expression is:

(5) $$\frac{P}{P_{u}}=\frac{S/d_{\mathrm{stud}}}{0.006+1.02\cdot S/d_{\mathrm{stud}}}$$

where *d*_stud_ represents the stud diameter, and its unit is mm.

Tong et al. [22] studied the effect of stud arrangement on the static behavior of stud connectors in high strength steel-UHPC composite beams, and the empirical expression for load-slip curves of this type of beam with single stud arrangement was obtained as:

(6) $$\frac{P}{P_{u}}=\frac{S/d_{\mathrm{stud}}}{0.0092+0.93\cdot S/d_{\mathrm{stud}}}$$

In Equation (1), the unit of *S* is inch, while in Equations (2)–(6), the unit of *S* is mm.

The test results indicated that the concrete type had an importance effect on the performance of stud connectors. Hence, the above load-slip formulas for NSC and UHPC specimen could not be well applied to the HSFRC specimens. In accordance with the previous research and the experiment results of HSFRC specimens in this study, an empirical prediction formula for the load-slip curves of the studs in steel-HSFRC composite beams was proposed. Next, the coefficients were derived by linear regression analysis. The formula is as follows:

(7) $$\frac{P}{P_{u}}=\frac{(5.664-0.0956\cdot d_{\mathrm{stud}})\cdot S}{1+(5.314-0.09116\cdot d_{\mathrm{stud}})\cdot S}$$

It was noted that in the fitting of Equation (7), only the test point with *P*/*P*_u_ less than 1 were used. Since the descending section of the curve had little significance for the static performance characterization of the stud connectors.

**Figure 8:**
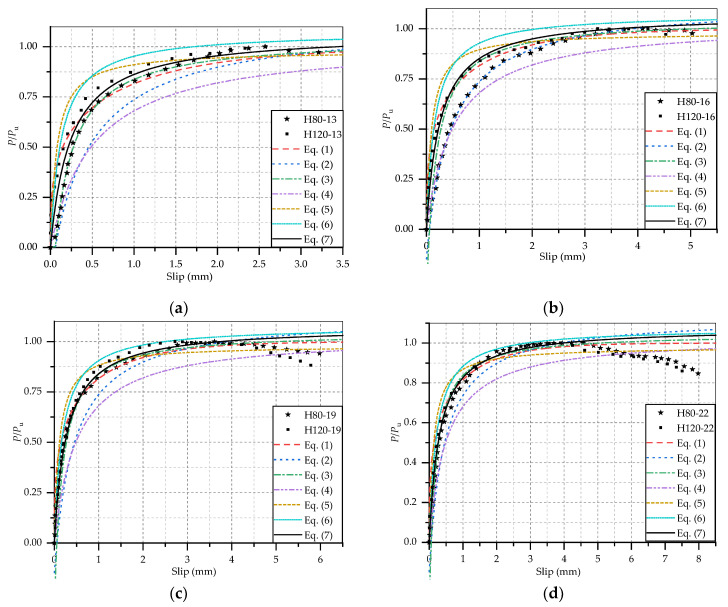
Comparison of experimental and calculated load-slip curves: (**a**) *d_stud_* = 13 mm; (**b**) *d_stud_* = 16 mm; (**c**) *d_stud_* = 19 mm; (**d**) *d_stud_* = 22 mm.

The experimental load-slip curves of HSFRC specimens and the load-slip curves calculated by Equations (1)–(7) are shown in Figure 8. It can be seen that the curves obtained by Equation (7) are generally closer to the test curves than the other equations. The comparison of some key points (*P*/*P*_u_ = 0.4, 0.6 0.8 and 1.0) obtained by test results and Equations (1)–(7) are listed in Table 6. In addition, the correlation coefficients between experimental and calculated data are also given in this table. The differences between the data calculated by Equation (7) and the test values of each HSFRC specimens are smaller, and the correlation coefficient of Equation (7) is the closest to 1. Therefore, Equation (7) could be well used in calculating the load-slip curves of stud connectors in steel-HSFRC composite beams.

### 4.2. Shear Capacity

The shear capacity of stud connectors is an important parameter in design of steel-concrete composite beams. Relevant codes in some mainstream regions have given several calculation methods of shear capacity of studs in steel-NSC composite beam. Many researchers have also developed some shear capacity prediction formulas for studs in different types of steel-concrete composite beam by push-out test.

Combined with the test data of 75 push-out specimens and the calculation model proposed by Ollgaard et al. [34], the Eurocode-4 [14] stipulates that the stud shear capacity should be calculated according to the following formula:

(8) $$P_{u}=0.29\alpha d^{2}\sqrt{f_{\mathrm{ck}}E_{c}}/\gamma_{v}\leq 0.8A_{s}f_{\mathrm{uk}}/\gamma_{v}$$

where *d* is the diameter of stud; *f*_ck_ is the standard compressive strength of concrete; *E*_c_ is the elasticity modulus of concrete; *γ*_v_ is the partial safety factor; *A*_s_ is the cross-sectional area of stud; *f*_uk_ is the standard tensile strength of stud; and *α* is the influence coefficient of stud aspect ratio which can be calculated by:

(9) $$\left\{ \begin{array}{ll} \alpha =0.2(\frac{h_{s}}{d}+1), & 3\leq \frac{h_{s}}{d}\leq 4\\ \alpha =1.0, & \frac{h_{s}}{d}>4 \end{array}\right.$$

where *h*_s_ is the height of stud.

In America, the code AASHTO LFRD [15] provide the following formula for calculating the nominal shear capacity of single stud connecters in steel-concrete composite beam:

(10) $$P_{u}=\varphi_{\mathrm{sc}}0.5A_{s}\sqrt{E_{c}f_{c}\prime }\leq \varphi_{\mathrm{sc}}A_{s}f_{u}$$

where *f*_c_′ is the compressive strength of concrete cylinder; *f*_u_ is the tensile strength of stud; and *φ*_sc_ is the resistance coefficient of stud which is equal to 0.85.

In the Chinese code GB50017-2017 [16], the shear capacity of cylindrical head welding stud connectors embedded in concrete is defined as:

(11) $$P_{u}=0.43A_{s}\sqrt{E_{c}f_{c}}\leq 0.7A_{s}f_{u}$$

where *f*_c_ is the cube compressive strength of concrete.

The formulas for shear capacity of studs given in above codes are mainly taken the strength of concrete crushing as shear capacity of studs in composite beams, and the tensile failure strength of stud shank is regard as the upper limit of stud shear capacity. These formulas only consider the concrete crush and stud fracture separately, while ignoring the interaction between stud connectors and concrete slabs. Hence, according to Equations (9)–(11), the shear capacity of studs with same dimension in NSC and HSFRC specimens are equal, which is not consistent with the actual test results. In view of this deficiency, Xue et al. [12] developed the following improved formula:

(12) $$P_{u}=\min\{0.43A_{s}\sqrt{E_{c}f_{c}},3\lambda f_{u}{\left(\frac{E_{c}}{E_{s}}\right)}^{0.4}{\left(\frac{f_{c}}{f_{u}}\right)}^{0.2}\}$$

where *λ* is the influence coefficient of stud aspect ratio and calculated as:

(13) $$\lambda =\left\{ \begin{array}{ll} 6-\frac{h_{s}}{1.05d}, & \frac{h_{s}}{d}\leq 5\\ 1, & 5<\frac{h_{s}}{d}\leq 7\\ \frac{h_{s}}{d}-6, & 7<\frac{h_{s}}{d} \end{array}\right.$$

Based on the test results of steel-UHPC with short studs, Shao et al. [21] proposed a stud strength formula which considered the contribution of local crushing concrete around the stud root. It is noted that the failure model of all specimens is stud fracture. The formula is as follows:

(14) $$P_{u}=(0.85+\frac{f_{c}}{f_{u}})A_{s}f_{u}/\gamma$$

where *γ* is resistance coefficient.

The above formulas were mainly used to calculate the shear capacity of the stud connectors embedded in NSC and UHPC, and they did not consider the concrete tensile strength. In fact, the tensile strength of HSFRC was significantly bigger than that of NSC due to the existence of fiber, which might influence the shear capacity of studs. Hence, a formula for calculating the stud shear capacity was proposed in this paper, which considered the concrete tensile strength and was suitable for studs embedded in different types of concrete. In this formula, the shear capacity of stud connectors was divided into the shear capacity of stud without constraint and the increasement of strength caused by concrete [36]. The shear capacity of stud without constraint was defined as a half of stud tensile capacity, and the increasement of strength was connected with the stud diameter, the compressive and tensile strength of concrete and the elastic modulus of concrete. Combined with the previous formulas and the influencing factors proposed in this study, the basic forms of the formula for the second part of shear capacity were given. Then the unknown coefficients in the formula were derived through linear regression analysis. Finally, the prediction formula for the shear capacity of stud in steel-concrete composite beam were proposed as:

(15) $$P_{u}=0.5A_{s}f_{u}+K[1+(\frac{f_{t}}{f_{u}}{)}^{0.5}](\frac{f_{c}}{f_{u}}{)}^{0.2}{\left(E_{c}d\right)}^{0.5}$$

where *f*_t_ is the tensile strength of concrete and K is a constant which is equal to 95.3.

Table 7 shows the comparisons of stud shear capacity between test results and calculation results. It is noted that to better compare the accuracy of the above prediction formulas, the resistance coefficient γ_v_ and γ are assumed to be equal to 1. It can be found that the prediction formulas in the three current codes were conservative and Xue et al. [12] overestimated the shear capacity. The formula proposed by Shao et al. was relatively accurate, but the error fluctuated a lot. It is clear that the result calculated by Equation (15) can agree better with the test results. Hence, Equation (15) can be well used to predict the shear capacity of studs in steel-NSC and steel-HSFRC composite beams.

**Figure 9:**
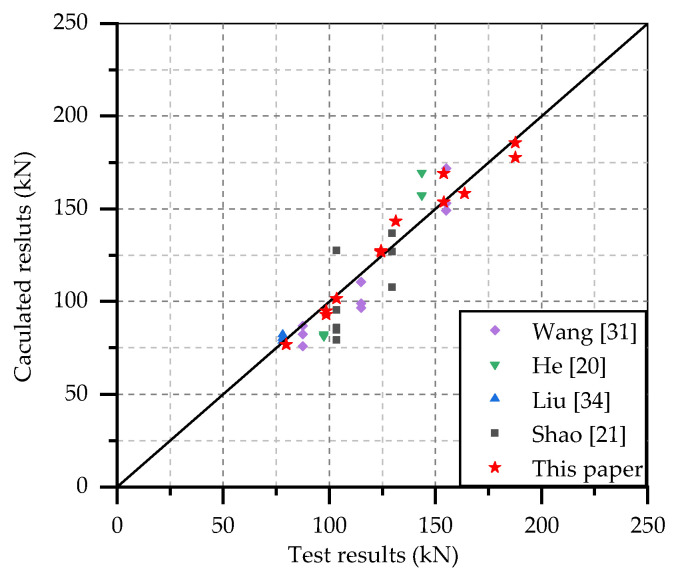
Comparison of test and calculated results of shear capacity in other literatures.

In order to verify the applicability of the shear capacity formula proposed in this paper, the experimental results in other literatures [20,21,33,37] were collected and compared with the calculated results by Equation (15), as shown in Figure 9. These studs are embedded in the NSC [33], FRC [20,37] and UHPC [21], respectively. It can be seen that the calculated results are in good agreement with the test results and the average error is 7.86%, which indicates that the formula has a good applicability for studs embedded in different type of concrete.

## 5. Conclusions

In this study, 12 push-out specimens with the test parameter of concrete type, concrete strength, stud diameter and stud height were tested to research the static performance of stud connectors in steel-NSC and steel-HSFRC composite beams. In accordance with the test results and the above analysis, the following major conclusions can be drawn:

(1) In the push-out tests, the steel-NSC and steel-HSFRC composite beams shown different failure modes. The failure modes of steel-NSC specimens were combine failure of concrete and studs, and those of steel-HSFRC specimens were stud failure. Some diagonal and splitting cracks occurred on the surfaces of the NSC slabs, and the number of cracks increased with stud diameter. However, there were no visible cracks on the HSFRC slabs. This indicated that the cracking resistance of steel-HSFRC composite beams was better than that of steel-NSC.
(2) The static performance of stud connectors would be influenced by the concrete type and the diameter and height of stud. In comparison with the studs embedded in NSC, the shear capacity and stiffness of studs embedded in HSFRC increased significantly but the ductility would decrease. With the diameter and height of stud increasing, the shear capacity and stiffness of studs increased while the ductility decreased. Among them, the influence of stud height was slight, which could be ignore when the stud diameter was reasonable.
(3) Based on the test results, the empirical formulas for the load-slip curve and shear capacity of stud connectors in steel-HSFRC composite beams were proposed. Thereinto, the formula for load-slip curve took the stud diameter into account, and the formula for shear capacity divided the shear capacity of studs into the shear capacity of stud without constraint and the increasement of strength caused by concrete. In addition, the influence of concrete tensile strength was considered in shear capacity formula. These two formulas were more consistent with the actual situation and could more accurately predict the static behavior of steel-HSFRC composite beams.

## Figures and Tables

**Table 1:** Details of the push-out specimen.

Specimen ID	Concrete Type	Stud Dimension		
		Diameter (mm)	Height (mm)	Aspect Ratio
N80-13	NSC	80	13	6.15
N80-16	NSC	80	16	5.00
N80-19	NSC	80	19	4.21
N80-22	NSC	80	22	3.64
H80-13	HSFRC	80	13	6.15
H80-16	HSFRC	80	16	5.00
H80-19	HSFRC	80	19	4.21
H80-22	HSFRC	80	22	3.64
H120-13	HSFRC	120	13	9.23
H120-16	HSFRC	120	16	7.50
H120-19	HSFRC	120	19	6.32
H120-22	HSFRC	120	22	5.45

*Note*: N and H represent NSC and HSFRC, respectively, and the number after them represent the height of stud. The numbers after “-” indicate the diameter of the stud. For instance, specimen H80-16 refers to the specimen with HSFRC and studs with height of 80 mm and diameter of 16 mm.

**Table 2:** The mixture proportions of concretes.

Component	Mix Quantity (kg/m^3^)	
	NSC	HSFRC
Cement P.O 52.5	444	687
Water	160	160
Broken stone 5~20 mm	1163	960
Sand 0~5 mm	626	517
Superplasticizer	4.4	7.61
Silica fume	-	75
Steel fiber (%)	-	156 (2%)

**Table 3:** Material property of concrete.

Concrete Type	Compressive Strength *f*_cu_ (MPa)	Tensile Strength *f*_tu_ (MPa)	Elastic Modulus *E*_c_ (GPa)
NSC	53.4	1.9	37.5
HSFRC	105.5	18.9	46.0

**Table 4:** Mechanical properties of studs.

Diameter (mm)	Yield Strength *f*_y_ (MPa)	Tensile Strength *f*_t_ (MPa)	Elastic Modulus *E*_s_ (GPa)
13	375	530	195
16	380	540	195
19	385	550	195
22	390	560	195

**Table 5:** Push-out test results.

Specimen	*P*_max_ (kN)	*P*_u_ (kN)	*S*_u_ (mm)	Slip at 0.2 mm		Slip at 2 mm		Failure Mode
				Load (kN)	*k*_1_ (kN/mm)	Load (kN)	*k*_2_ (kN/mm)	
N80-13	613.76	76.72	4.89	35.33	176.65	67.97	33.98	Combine failure
N80-16	812.16	101.52	6.09	26.51	132.57	83.55	41.78	Combine failure
N80-19	1145.84	143.23	7.61	51.05	255.24	120.67	60.33	Combine failure
N80-22	1266.72	158.34	9.76	46.35	231.74	128.69	64.34	Combine failure
H80-13	759.12	93.01	3.21	53.00	265.01	92.46	44.85	Stud failure
H80-16	1014.08	126.76	5.03	32.74	163.68	111.76	55.88	Stud failure
H80-19	1228.64	153.58	5.98	75.68	378.41	142.56	71.28	Stud failure
H80-22	1421.28	177.66	7.98	65.40	327.00	170.23	85.11	Stud failure
H120-13	744.08	94.89	2.58	34.47	172.33	89.70	46.23	Stud failure
H120-16	1018.8	127.35	4.53	63.55	317.77	116.95	58.48	Stud failure
H120-19	1352.08	169.01	5.78	77.58	387.91	164.54	82.27	Stud failure
H120-22	1485.92	185.74	7.51	87.44	437.18	174.08	87.04	Stud failure

*Note*: *P_max_* represent the ultimate load of push-out specimen; *P*_u_ represents the shear capacity of per stud; *S*_u_ represents the ultimate interfacial slip; Load represents the load on per stud; and *k*_1_ and *k*_2_ represent the shear stiffness of stud when the slip at 0.2 and 2 mm, respectively.

**Table 6:** Comparison of load-slip curves obtained by test results and prediction formula.

Specimen	*P*/*P*_u_, Exp.	*S* (mm)	*P*/*P*_u_, Equation						
			Equation (1)	Equation (2)	Equation (3)	Equation (4)	Equation (5)	Equation (6)	Equation (7)
H80-13	0.4	0.207	0.550	0.258	0.405	0.295	0.716	0.663	0.493
	0.6	0.367	0.643	0.429	0.594	0.429	0.811	0.796	0.644
	0.8	0.827	0.784	0.683	0.801	0.635	0.897	0.931	0.828
	1.0	2.570	0.948	0.942	0.957	0.859	0.952	1.024	0.979
H120-13	0.4	0.095	0.441	0.037	0.070	0.131	0.485	0.396	0.302
	0.6	0.248	0.578	0.309	0.467	0.335	0.749	0.708	0.542
	0.8	0.596	0.726	0.584	0.728	0.553	0.869	0.884	0.761
	1.0	2.575	0.949	0.942	0.957	0.859	0.952	1.024	0.978
H80-16	0.4	0.333	0.626	0.399	0.564	0.405	0.764	0.729	0.603
	0.6	0.608	0.730	0.590	0.733	0.558	0.849	0.853	0.752
	0.8	1.234	0.850	0.791	0.872	0.727	0.911	0.953	0.886
	1.0	4.090	0.983	1.005	0.989	0.916	0.958	1.035	1.008
H120-16	0.4	0.121	0.472	0.125	0.221	0.196	0.551	0.466	0.341
	0.6	0.322	0.621	0.388	0.553	0.396	0.759	0.721	0.594
	0.8	0.823	0.783	0.681	0.800	0.634	0.863	0.875	0.815
	1.0	4.195	0.984	1.008	0.991	0.918	0.955	1.029	1.009
H80-19	0.4	0.125	0.477	0.132	0.232	0.201	0.518	0.429	0.331
	0.6	0.330	0.625	0.396	0.561	0.402	0.732	0.685	0.582
	0.8	0.900	0.798	0.707	0.818	0.655	0.872	0.889	0.820
	1.0	3.610	0.976	0.990	0.982	0.902	0.955	1.030	0.997
H120-19	0.4	0.163	0.514	0.195	0.322	0.248	0.582	0.499	0.395
	0.6	0.335	0.627	0.400	0.566	0.406	0.735	0.689	0.586
	0.8	0.723	0.760	0.643	0.773	0.602	0.849	0.853	0.775
	1.0	2.720	0.954	0.951	0.962	0.867	0.942	1.006	0.974
H80-22	0.4	0.217	0.557	0.271	0.422	0.305	0.614	0.537	0.450
	0.6	0.442	0.674	0.488	0.649	0.476	0.758	0.721	0.639
	0.8	1.062	0.826	0.753	0.848	0.694	0.874	0.892	0.838
	1.0	4.560	0.988	1.017	0.995	0.964	0.9533	1.026	1.009
H120-22	0.4	0.156	0.508	0.184	0.307	0.240	0.536	0.449	0.367
	0.6	0.329	0.624	0.395	0.560	0.402	0.704	0.647	0.561
	0.8	0.881	0.794	0.701	0.814	0.650	0.855	0.862	0.801
	1.0	3.895	0.980	1.000	0.986	0.910	0.949	1.018	0.999
Correlation coefficient	-	-	0.940	0.890	0.906	0.907	0.895	0.933	0.982

**Table 7:** Comparisons of test and calculation results of stud shear capacity.

Specimen	Test (kN)	Equation (8)/Test	Equation (10)/Test	Equation (11)/Test	Equation (12)/Test	Equation (14)/Test	Equation (15)/Test
N80-13	76.72	0.73	0.78	0.64	0.90	0.87	1.04
N80-16	101.52	0.86	0.91	0.75	1.21	1.01	1.02
N80-19	143.23	0.87	0.93	0.76	1.20	1.03	0.92
N80-22	158.34	1.08	1.14	0.94	1.46	1.27	1.03
H80-13	93.01	0.61	0.64	0.53	0.92	0.79	1.06
H80-16	126.76	0.69	0.73	0.60	1.29	0.90	0.98
H80-19	153.58	0.81	0.86	0.71	1.75	1.06	1.00
H80-22	177.66	0.96	1.02	0.84	2.03	1.24	1.06
H120-13	94.89	0.59	0.63	0.52	1.33	0.78	1.04
H120-16	127.35	0.68	0.72	0.60	1.50	0.89	0.98
H120-19	169.01	0.74	0.78	0.65	1.12	0.96	0.91
H120-22	185.74	0.92	0.97	0.80	1.38	1.19	1.01
Average	-	0.79	0.84	0.70	1.34	1.00	1.00
Standard deviation	-	0.15	0.16	0.13	0.32	0.17	0.05

## Data Availability

Data is contained within the article.

## References

[1] Chen J., Zhang H.P., Yu Q.Q. (2019). Static and fatigue Behaviour of steel-concrete composite beams with corroded studs. J. Steel Res..

[2] Lowe D., Roy K., Das R., Clifron C.G., Lim J.B. (2020). Full scale experiments on splitting behaviour of concrete slabs in steel concrete composite beams with shear stud connection. Structures.

[3] Wang J.Y., Guo J.Y., Jia L.J., Chen S.M., Dong Y. (2017). Push-out tests of demountable headed stud shear connectors in steel-UHPC composite structures. Compos. Struct..

[4] Hosseini S.M., Mashiri F., Mirza O. (2020). Research and developments on strength and durability prediction of composite beams utilising bolted shear connectors (Review). Eng. Fail. Anal..

[5] Shen M.H., Chung K.F. (2017). Structural behaviour of stud shear connections with solid and composite slabs under co-existing shear and tension forces. Structures.

[6] Oehlers D.J., Coughlan C.G. (1986). The shear stiffness of stud shear connections in composite beams. J. Steel Res..

[7] Han Q.H., Wang Y.H., Xu J., Xing Y. (2015). Static behaviour of stud shear connectors in elastic concrete–steel composite beams. J. Steel Res..

[8] Molkens T., Dobrić J., Rossi B. (2019). Shear resistance of headed shear studs welded on welded plates in composite floors. Eng. Struct..

[9] Buttry K.E. (1965). Behaviour of Stud Shear Connectors in Lightweight and Normal-Weight Concrete. Ph.D. Thesis.

[10] Ding F.X., Yin G.A., Wang H.B., Wang L., Guo Q. (2017). Static behaviour of stud connectors in bi-direction push-off tests. Thin Wall. Struct..

[11] Wang J.F., Zhang A.P., Wang W.H. (2020). Effects of stud height on shear behaviour of stud connectors. J. Zhejiang Univ..

[12] Xue W.C., Ding M., Wang H., Luo Z.W. (2008). Static behaviour and theoretical model of stud connectors. J. Bridge Eng..

[13] Xue C., Liu Y., Yu Z. (2012). Static behaviour of multi-stud shear connectors for steel-concrete composite bridge. J. Constr. Steel Res..

[14] (2004). EN 1994-1-1. Eurocode4: Design of Composite Steel and Concrete Structures.

[15] (2017). AASHTO LRFDUS-2017. AASHTO LRFD Bridge Design Specifications.

[16] (2017). GB 50017-2017, Standard for Design of Steel Structures.

[17] Wang J.Q., Xu Q.Z., Yao Y.M., Qi J.N., Xiu H.L. (2018). Static behaviour of grouped large headed stud-UHPC shear connectors in composite structures. Compos. Struct..

[18] Zhu L., Wang J.J., Li X., Tang L., Yu B.Y. (2020). Experimental and numerical study of curved SFRC and ECC composite beams with various connectors. Thin Wall. Struct..

[19] Zhang Y.J., Liu A.R., Chen B.C., Zhang J.P., Pi Y.L., Bradford M.A. (2020). Experimental and numerical study of shear connection in composite beams of steel and steel-fiber reinforced concrete. Eng. Struct..

[20] He Y.L., Guo S.J., Wang L.C., Yang Y., Xiang Y.Q. (2020). Experimental and numerical analysis of grouped stud shear connectors embedded in HFRC. Constr. Build. Mater..

[21] Shao X.D., Li M., Cao J.H., He G., Chen Y.B., Zhao X.D. (2021). Experimental research and theoretical analysis on shear performance of short headed studs embedded in UHPC. China J. Highw. Transp..

[22] Tong L.W., Chen L.H., Wen M., Xu C. (2020). Static behaviour of stud shear connectors in high-strength-steel–UHPC composite beams. Eng. Struct..

[23] Wang J.Q., Qi J.N., Tong T., Xu Q.Z., Xiu H.L. (2019). Static behaviour of large stud shear connectors in steel-UHPC composite structures. Eng. Struct..

[24] Wang Z., Nie X., Fan J.S., Lu X.Y., Ding R. (2019). Experimental and numerical investigation of the interfacial properties of non-steam-cured UHPC-steel composite beams. Constr. Build. Mater..

[25] Banjara N.K., Ramanjaneyulu K. (2019). Experimental and numerical study on behaviour of HSFRC overlay strip strengthened flexural deficient RC beams. Eng. Struct..

[26] Ayub T., Shafiq N., Khan S.U. (2016). Compressive Stress-Strain Behaviour of HSFRC Reinforced with Basalt Fibers. J. Mater. Civil Eng..

[27] Golewski G.L. (2020). Changes in the fracture toughness under mode II loading of low calcium fly ash (LCFA) concrete depending on ages. Materials.

[28] Luccioni B., Isla F., Codina R., Ambrosini D., Zerbino R., Giaccio G., Torrijos M.C. (2018). Experimental and numerical analysis of blast response of High Strength Fiber Reinforced Concrete slabs. Eng. Struct..

[29] Algassem O., Li Y., Aoude H. (2019). Ability of steel fibers to enhance the shear and flexural behaviour of high-strength concrete beams subjected to blast loads. Eng. Struct..

[30] (2010). GB/T 11263-2010, Hot-Rolled H and Cut T Section Steel.

[31] (2009). CECS 13: 2009. Standard Test Methods for Fiber Reinforced Concrete.

[32] Qi J.N., Wang J.Q., Li M., Chen L. (2017). Shear capacity of stud shear connectors with initial damage: Experiment, FEM model and theoretical formulation. Steel Compos. Struct..

[33] Wang W.H. (2018). Experimental and Analytical Study on Shear Properties of Headed Stud Connector. Master’s Thesis.

[34] Ollgaard J.G., Sluttter R.G., Fisher J.W. (1971). Shear strength of stud connectors in lightweight and normal-weight concrete. AISC Eng. J..

[35] An L., Cederwall K. (1996). Push-out tests on studs in high strength and normal strength concrete. J. Constr. Steel Res..

[36] Hegger J., Sedlacek G., Doinghaus P., Trumpf H. (2001). Investigations on the ductility of shear connectors when using high-strength steel and high-strength concrete. International Symposium on Connections between Steel and Concrete.

[37] Liu Y., Zhang Q., Bao Y., Bu Y. (2018). Static and fatigue push-out tests of short headed shear studs embedded in Engineered Cementitious Composites (ECC). Eng. Struct..

